# Huge upconversion luminescence enhancement by a cascade optical field modulation strategy facilitating selective multispectral narrow-band near-infrared photodetection

**DOI:** 10.1038/s41377-020-00418-0

**Published:** 2020-10-30

**Authors:** Yanan Ji, Wen Xu, Nan Ding, Haitao Yang, Hongwei Song, Qingyun Liu, Hans Ågren, Jerker Widengren, Haichun Liu

**Affiliations:** 1 State Key Laboratory of Integrated Optoelectronics, College of Electronic Science and Engineering, Jilin University, 130012 Changchun, China; 2 Department of Theoretical Chemistry and Biology, KTH Royal Institute of Technology, SE-106 91, Stockholm, Sweden; 3 Department of Applied Physics, KTH Royal Institute of Technology, SE-106 91, Stockholm, Sweden

**Keywords:** Nanoparticles, Optoelectronic devices and components

## Abstract

Since selective detection of multiple narrow spectral bands in the near-infrared (NIR) region still poses a fundamental challenge, we have, in this work, developed NIR photodetectors (PDs) using photon upconversion nanocrystals (UCNCs) combined with perovskite films. To conquer the relatively high pumping threshold of UCNCs, we designed a novel cascade optical field modulation strategy to boost upconversion luminescence (UCL) by cascading the superlensing effect of dielectric microlens arrays and the plasmonic effect of gold nanorods, which readily leads to a UCL enhancement by more than four orders of magnitude under weak light irradiation. By accommodating multiple optically active lanthanide ions in a core-shell-shell hierarchical architecture, developed PDs on top of this structure can detect three well-separated narrow bands in the NIR region, i.e., those centered at 808, 980, and 1540 nm. Due to the large UCL enhancement, the obtained PDs demonstrate extremely high responsivities of 30.73, 23.15, and 12.20 A W^−1^ and detectivities of 5.36, 3.45, and 1.91 × 10^11^ Jones for 808, 980, and 1540 nm light detection, respectively, together with short response times in the range of 80–120 ms. Moreover, we demonstrate for the first time that the response to the excitation modulation frequency of a PD can be employed to discriminate the incident light wavelength. We believe that our work provides novel insight for developing NIR PDs and that it can spur the development of other applications using upconversion nanotechnology.

## Cascade amplified upconversion luminescence: Applied in narrow band NIR photodetection

Selective detection of multiple narrow spectral bands in the near-infrared (NIR) region is still a challenge. Recently, Hongwei Song and Wen Xu at Jilin University/China, Haichun Liu at KTH Royal Institute of Technology/Sweden, and their co-workers have successfully fabricated a novel multiple NIR bands photo-detectors (PDs) by combining multiple-excitation-bands core-shell upconversion nanocrystals (UCNCs) with MAPbI_3_ perovskite photoelectric conversion layer. Through a cascade optical field modulation strategy, a combination of microlenses and gold plasmon nanocrystals, the emission intensity of the UCNCs and the photoelectric signal of the PDs can be enhanced four orders of magnitude. Moreover, the excitation frequency of the PD has been employed to discriminate the wavelength of incident light for the first time. This work provides a novel insight for developing multiple bands NIR PDs, and for applications of upconversion nanotechnology.

## Introduction

Narrow-band near infrared (NIR) photodetectors (PDs) capable of simultaneously detecting light in multispectral bands, e.g., in the NIR I and NIR II regions, are attracting substantial attention in diverse areas, including biological analysis, multicolor bioimaging/sensing, and encrypted communications^1–4^. Currently, the major technologies for multispectral NIR PDs concentrate on integrating multiple NIR-response materials with different bandgaps, e.g., HgCdTe (MCT), quantum wells, superlattices, two-dimensional metal chalcogenides and lanthanide upconversion nanocrystals (UCNCs)^5–12^. Among other materials, UCNCs, due to their unique two-photon or multiphoton excitation nature, nontoxic characteristics and low preparation cost^13–18^, have emerged as a superior solution by converting NIR photons into easily detectable visible photons. However, they have a relatively high pumping threshold to realize detectable upconversion luminescence (UCL), which originates from the lower absorption cross section of *4f*^*n*^*-4f*^*n*^ transitions of rare earth (RE) ions and lower luminescent quantum efficiency of UCNCs because of the anti-Stokes nature and poses a fundamental limitation for weak NIR light detection in photoelectric devices^19^.

In the past, a few approaches have been explored to boost UCL and decrease the pumping threshold of UCNCs, e.g., nanocrystal surface passivation, photonic crystal engineering, plasmon/organic antennas, and superlensing effects^19,20^. Among other techniques, utilizing the localized surface plasmon resonance (LSPR) of noble-metal nanostructures and the superlensing effect of dielectric optical microstructures can serve as two efficient strategies to take advantage of the highly nonlinear response of UCNCs to excitation intensity^21–26^. Although significant UCL enhancements (up to four orders of magnitude in some extreme cases) can potentially be achieved by using these optical amplifiers^27^, the UCL enhancement is strictly limited by localization of the hotspot induced in the light field, which is typically much smaller than the dimensions of the UCNCs. A large UCL enhancement requires delicately designed plasmonic nanostructures or dielectric optical microstructures, which would require obstructive fabrication technology and cost.

Wisely designing the hierarchical structure of UCNCs to integrate multiple types of lanthanide ions into single nanoparticles can potentially achieve the detection of multispectral bands. Nevertheless, finding a practical way to separate detectable channels to decode more information is still very challenging in such PDs if one wants to avoid complicated optical system design and integration, e.g., employing additional spectral components.

In this work, to overcome the shortcomings of individual amplifiers, we explored a novel cascade optical field modulation strategy integrating the superlensing effect of polymeric microlens arrays (MLAs) and the plasmonic effect of gold nanorods (Au NRs) to boost UCL. This cascade modulation strategy readily led to a UCL enhancement of more than four orders of magnitude. Such huge UCL enhancement enabled us to break through the bottleneck of UCNC-based photodetection technology and build high-performance NIR PDs with extremely high responsivity and detectivity. We designed and synthesized multi-wavelength responsive core-shell-shell (CSS)-structured UCNCs that emit visible light under excitation at 808, 980, or 1540 nm and constructed NIR PDs on top. Realizing that each UCNC constitutes an information-rich kinetic system exhibiting characteristic responses to optical signals in the temporal and frequency domains of different excitation wavelengths^28,29^, we exploited the possibility of separating the channels of multi-wavelength photodetection to implement selective detection. We proved that the modulation frequency response can be used to distinguish the detected wavelengths well. In addition, the UCL kinetics of the UCNCs were also optimized by manipulating the concentrations of lanthanide dopants, whereby short response times of 80–120 ms for the final PDs were achieved.

## Results

### Multiwavelength-responsive UCNCs

**Fig. 1:**
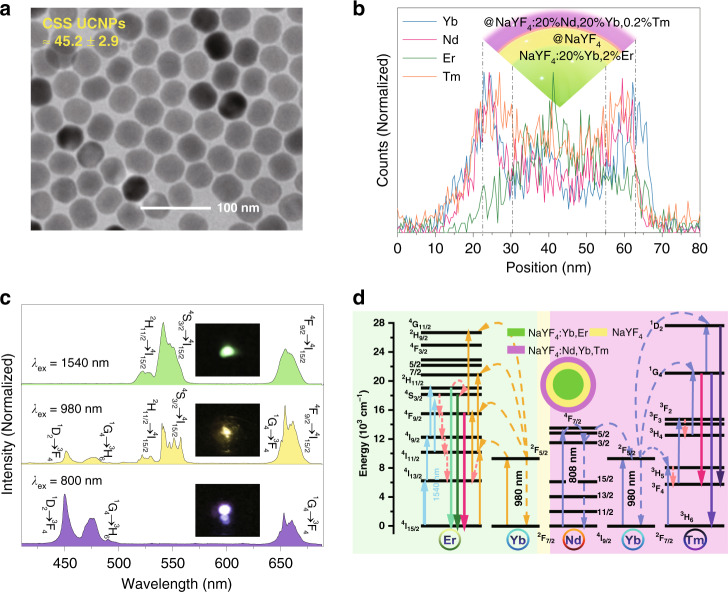
Multi-wavelength-responsive UCNCs. **a** TEM image of NaYF_4_:20% Yb^3+^, 2%Er^3+^@NaYF_4_@NaYF_4_:20% Yb^3+^, 20% Nd^3+^, 0.2% Tm^3+^ CSS UCNCs. **b** Line-scan elemental mapping curves of a single NaYF_4_:20% Yb^3+^, 2% Er^3+^@NaYF_4_@NaYF_4_:20% Yb^3+^, 20% Nd^3+^, 0.2% Tm^3+^ CSS NC. **c** Upconversion emission spectra of CSS UCNCs recorded under 808, 980 and 1540 nm laser excitation. (Inset) Corresponding photos of the CSS UCNC films upon illumination with three kinds of lasers. **d** Energy-transfer upconversion processes in Nd^3+^-, Yb^3+^-, Er^3+^-, and Tm^3+^-codoped CSS UCNCs

CSS NaYF_4_:Yb^3+^, Er^3+^@NaYF_4_@NaYF_4_:Yb^3+^, Nd^3+^, Tm^3+^ UCNCs were synthesized using a standard solvothermal method, and they could respond to 808, 980, and 1540 nm light and emit visible light^23,30,31^. Figure 1a shows a transmission electron microscopy (TEM) image of the synthesized CSS UCNCs. Monodispersed and homogeneous NCs were fabricated with a diameter of 45.0 ± 2.9 nm. The diameter of the corresponding core NaYF_4_:Yb^3+^, Er^3+^ NCs was determined to be 15.0 ± 1.8 nm, and the thicknesses of the NaYF_4_ and NaYF_4_:Yb^3+^, Nd^3+^, Tm^3+^ shells were calculated to be 5.3 ± 0.3 nm and 9.8 ± 0.3 nm, respectively, based on the TEM data of the core and the intermediate core-shell NCs (Supplementary Fig. S1). Line-scan elemental mapping curves of single CSS NCs are displayed in Fig. 1b. Consistently, the element mapping analysis shown in Supplementary Fig. S2 further demonstrates the successful construction of the CSS structure of UCNCs. All the UCNCs have a high crystallinity and a hexagonal phase, as shown in Supplementary Fig. S3. These CSS UCNCs can emit the typical upconversion emission of Er^3+^ ions (from the inner core), the mixed emission of Er^3+^ ions (inner core) and Tm^3+^ (from the outer shell), and the emission of Tm^3+^ (outer shell) under illumination at 1540, 980, and 808 nm, thereby exhibiting greenish, yellowish, and blue-violet colors, respectively, as shown in Fig. 1c. The green emission at 525/545 nm can be attributed to the ^2^H_11/2_/^4^S_3/2_ → ^4^I_15/2_ transitions, and the red emission at 650 nm can be attributed to the ^4^F_9/2_ → ^4^I_15/2_ transition of Er^3+^ ions. Tm^3+^ ions contribute to the blue-violet and red emissions through transitions from higher-lying ^1^D_2_ and ^1^G_4_ states to the lower ^3^F_4_ and ^3^H_6_ states. The photon upconversion pathways under different excitation wavelengths have been well studied previously and are depicted in Fig. 1d. Briefly, 1540 nm light can directly pump Er^3+^ ions and generate visible emissions through multiple excited state absorption processes^32^. In addition, 980 nm light can resonantly excite Yb^3+^ ions and further pump Er^3+^ and Tm^3+^ ions through nonradiative energy transfers, resulting in yellowish upconversion emissions. Under 808 nm excitation, Nd^3+^ ions in the outer shell dominate the absorption and migrate the energy to Yb^3+^ ions and subsequently to Tm^3+^ ions, giving rise to blue-violet and red emissions^33^. The inert layer of NaYF_4_ acts to prevent cross talk between the two emissive areas (the core and the outer shell)^34^. The outermost layer is not optimal, as Nd^3+^ has multiple higher energy levels that may effectively accept the energy from excited Tm^3+^ ions. Possible back energy transfer mechanisms from Tm^3+^ to Nd^3+^ under 808 nm excitation are illustrated in Supplementary Fig. S4. Meanwhile, there is likely cross relaxation between Nd^3+^ ions in heavily doped nanoparticles (Supplementary Fig. S4). The Nd^3+^-sensitized and Tm^3+^-emitting regions can be further optimized by separating Nd^3+^ and Tm^3+^ in different layers and controlling the Nd^3+^ concentration quenching.

### Cascade amplifiers for UCNCs

**Fig. 2:**
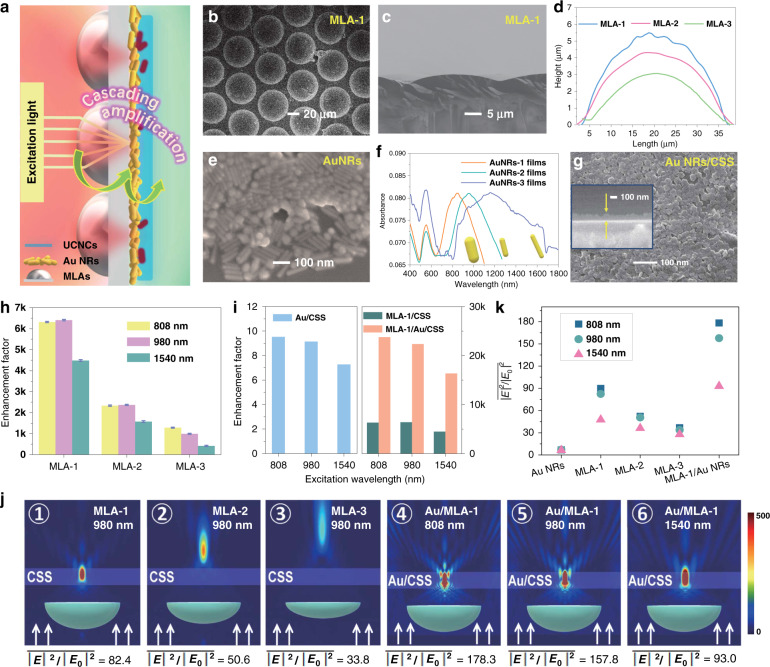
Cascading upconversion amplification effect based on the MLA/Au NR/CSS UCNC hybrid structure. **a**, Schematic illustration of the cascading amplification strategy for UCNCs. **b**, **c** Top-view (**b**) and cross-section (**c**) SEM images of MLA-1. **d** AFM characterization of the heights of MLA-1, MLA-2, and MLA-3. **e** SEM image of the Au NR film. **f** Absorption spectra of Au NR films with different aspect ratios. **g** Top-view SEM image of Au NR/CSS UCNC hybrids. (Inset) Cross-sectional SEM image of Au NR/CSS UCNC hybrids. **h** Enhancement factors for three kinds of MLAs under excitation at 808, 980, and 1540 nm in MLA/CSS UCNC composites at an excitation power density of 2 mW cm^−2^. **i** Enhancement factors for Au NR/CSS, MLA-1/CSS, and MLA-1/Au NR/CSS composites under 808, 980, and 1540 nm excitation. **j** Simulated electric field intensity distribution of MLA-1, MLA-2, MLA-3 and MLA-1/Au NR structures. Note that 808, 980 and 1540 nm plane wave light sources are used for excitation. **k** The calculated average electric field intensity $\overline {\left| E \right|^2/\left| {E_0} \right|^2}$ of Au NRs, MLAs, and MLA-1/Au NRs with incident wavelengths of 808, 980, and 1540 nm, respectively

Next, we exploited a cascade optical field modulation strategy for UCNCs by coupling the superlensing effect of microlens arrays (MLAs) and the plasmonic effect of Au NRs, as illustrated in Fig. 2a. The adaption of this cascading strategy was motivated by the consideration that MLAs can effectively confine far-field propagating incident light to a highly localized hotspot at the micrometer scale, while the Au NRs can tailor the photoelectric field spatially more precisely at the nanometer scale^35,36^. Such a synergistic field tuning effect cannot be achieved solely using routine MLAs or Au NRs.

Three polymeric MLAs (MLA-1, MLA-2, MLA-3) provided by a commercial company were tested. The MLAs consist of abundant hemisphere-like microlens units, as shown in the top-view and cross-section scanning electron microscopy (SEM) images of the MLA-1 sample (Fig. 2b, c). Atomic force microscopy (AFM) measurements of the MLAs demonstrate that the three MLAs have similar diameters of 35 µm but different heights of 5.5, 4.3, and 3.0 µm for MLA-1, MLA-2, and MLA-3, respectively (Fig. 2d). Gold NRs with three aspect ratios were prepared by a seed-mediated growth method (Supplementary Fig. S5a–c). Their LSPR peak maxima are 809, 989, and 1152 nm, respectively (Supplementary Fig. S5d). The scanning electron microscopy (SEM) image in Fig. 2e presents the plasmonic film of assembled Au NRs with a length of 120 nm and diameter of 20 nm. As revealed in Fig. 2f, after Au NRs are assembled, their LSPRs are broadened owing to the plasmonic coupling among Au NRs and overlap with the absorptions of Nd^3+^ at 808 nm, Yb^3+^ at 980 nm, and Er^3+^ at 1540 nm^26^.

The UCL enhancement effects of solo MLAs or Au NRs were first quantified by characterizing and comparing the photoluminescence output of UCNC and Au NR/UCNC films on MLAs or control substrates. All films were prepared using the same protocol, and the amount of UCNCs, their number density on the surface, and the thickness of the UCNC layer were carefully controlled to ensure fair comparisons between different groups. When MLAs were used as the substrate, the UCNCs or Au NRs/UCNCs covered the planar side of the MLAs (Fig. 2a). Figure 2g presents top-view and cross-sectional SEM images of the Au NR/UCNCs film deposited on a MLA substrate, indicating that the Au NR/UCNCs have a total thickness of approximately 110 nm. Detailed characterization by AFM shows that the thicknesses of the layers of Au NRs and UCNCs are approximately 65 and 45 nm, respectively (Supplementary Fig. S6). This suggests that two to three layers of Au NRs and a monolayer of UCNCs rested on the MLA substrate. Figure 2h presents the UCL enhancement factors in MLA-1/CSS, MLA-2/CSS, and MLA-3/CSS UCNC hybrids relative to the pristine CSS UCNCs under illumination with 808, 980, and 1540 nm light, where it can be seen that very prominent UCL enhancements have been obtained. In particular, for MLA-1, more than three orders of magnitude UCL improvement, 6300-, 6400-, and 4500-fold for illumination wavelengths of 808, 980, and 1540 nm, respectively, were observed. In contrast, UCL enhancements of 7–9.5-fold were found in Au NR/UCNC films, as shown in Fig. 2i (left panel).

Subsequently, the synergistic effect of MLAs and Au NRs was characterized. The most prominent MLA, MLA-1, was selected as the substrate for the deposition of Au NRs and UCNCs. Amazingly, after the superlensing effect of MLA-1 and the plasmonic effect of Au NRs were cascaded, the UCL enhancement factors are as high as 2.4×10^4^-, 2.2×10^4^-, and 1.6×10^4^-fold for 808, 980, and 1540 nm illumination, respectively (Fig. 2i, Right panel). It is worth mentioning that the excitation of Au NRs is dependent on the polarization of the incident light. Linearly polarized light can only excite the small portion of the Au NRs aligned well with the incident polarization, leading to efficiency loss. This might be improved by using polarization-insensitive Au nanoparticles^37^. The UCL decay curves for the ^4^S_3/2_ → ^4^I_15/2_ and ^4^F_9/2_ → ^4^I_15/2_ transitions of the Er^3+^ ions and the ^1^G_4_ → ^3^H_6_ transition of the Tm^3+^ ions were also measured under 980 nm excitation, as shown in Figure S7. The lifetimes of the MLA/UCNC and MLA-1/Au NR/UCNC CSS hybrids remain unchanged. The plasmon-induced luminescent enhancement can be classified into two categories: one is the interaction of LSPR with the emission electromagnetic field, resulting in the improved radiative transition rate of emitters, and the other is the interaction of LSPR with the excitation electromagnetic field, leading to the enhancement of localized excitation field strength without changing the radiative transition rate of emitters. Herein, the cases fall into the second category because the plasmon resonance wavelengths of Au NRs are located in the NIR region, matching the excitation wavelengths of UCNCs (as shown in Fig. 2f and Supplementary Fig. S5). These results suggest that the largely enhanced UCL from the UCNCs can be mainly ascribed to light field modulation rather than fundamental alterations in the optical transitions of lanthanide ions. To better understand the experimental results, some local optical field simulations using three-dimensional finite-difference time-domain (3D-FDTD) formulations were performed to identify the key factors in the UCL amplification. The influence of the MLA geometries was first studied. As indicated in Fig. 2j (j1-j3), under polarized plane-wave excitation at 980 nm, all three MLAs confine the incident light from the far field to a localized hotspot and thus largely increase the light intensity. Interestingly, the axial position of the hotspot significantly changes for different MLAs. It focuses on the UCNCs with stronger amplified optical field intensity around 82.4 folds in MLA-1, and is beneficial for effective coupling between UCNCs and MLAs, but deviates from the UCNCs in MLA-2 and MLA-3, implying that the height of the hemispheres in MLAs plays a critical role in the optical field distribution. Similar results were obtained for two other illumination wavelengths, 808 and 1540 nm, as shown in Supplementary Fig. S8. These simulated results are consistent with experimental observations that MLA-1 can obtain the most significant UCL enhancement for all three wavelengths, implying that the height of the hemispheres in MLAs is a crucial parameter for the given aperture of 35 µm. In addition, with increasing excitation wavelength from 808 nm to 1540 nm, the optical field strength in the hotspot gradually decreases (Supplementary Fig. S8a–c), which may result in illumination wavelength-induced deviation in the UCL enhancement. The LSPR effect of a film with assembled Au NRs (randomly distributed) was also simulated, as shown in Supplementary Fig. S8d, where average electric field enhancements of 6.9-, 7.2-, and 6.4-fold for 808, 980, and 1540 nm light were found, respectively. Next, we studied the cascade optical field modulation by the superlensing and plasmonic effects by simulations. The optical field distributions of MLA-1/Au NR-1, MLA-1/Au NR-2, and MLA-1/Au NR-3 hybrids under illumination with 808, 980, and 1540 nm light were calculated, as shown in Fig. 2j (j4-j6). The theoretical average electric field intensities $\overline {\left| E \right|^2/\left| {E_0} \right|^2}$ of Au NRs, MLAs, and MLA-1/Au NRs are summarized in Fig. 2k. As seen, through the cascade optical field modulation, the optical field strengths at 808, 980, and 1540 nm can be significantly boosted by 178.3-, 157.8-, and 93.0-fold by MLA-1/Au NRs, respectively, compared to 89.9-, 82.4-, and 47.7-fold for pristine MLA-1. Generally, UCL enhancement is proportional to $\left| E \right|^{2n}$, where *n* represents the number of excitation photons required to generate one upconverted photon. For the studied upconversion emission lines originating from two- or three-photon processes (Fig. 1d), *n* typically varies between 1.0 and 3.0, subject to upconversion saturation^26,32–34^. Thus, the excitation light intensity amplification through the joint action of the superlensing and plasmonic effects can potentially lead to UCL enhancement by four orders of magnitude for the utilized UCNCs^20^. Considering the finite size of the UCNCs (45 nm) and the localization of the plasmonic field generated by the Au NRs, only part of each nanocrystal was located within the hotspots. This can explain why the experimentally observed UCL enhancement is less than that predicted by simulations. In summary, these simulated results qualitatively approximate the experimentally observed UCL enhancements, and they together support the validity of the proposed cascade optical field modulation strategy for UCL. To further reveal the essential UCL enhancement, the UCL quantum yields of different devices were characterized under the same excitation power density of 1 W cm^−2^ 980 nm light. The quantum yields of UCNCs, AuNR/UCNCs, MLA/UCNCs and MLA/Au NR/UCNCs were determined to be 0.83%, 0.30%, 1.60%, and 1.86%, respectively. Because of nonradiative energy transfer from UCNCs to Au NRs (Supplementary Fig. S7)^38^, the quantum yield of Au NR/UCNCs is lower than that of pristine UCNCs. In contrast, the quantum yields of MLA/UCNCs and MLA/Au NR/UCNCs both improve upon that of pristine UCNCs. This can be mainly attributed to the excitation field enhancement effect rather than the Purcell effect, according to the variation in luminescent lifetimes. The quantum yield for UCL depends on the excitation power density. The improved excitation power density leads to improved quantum yield if the pump saturation effect does not occur^39^.

### Selective multispectral narrow-band NIR photodetection

**Fig. 3:**
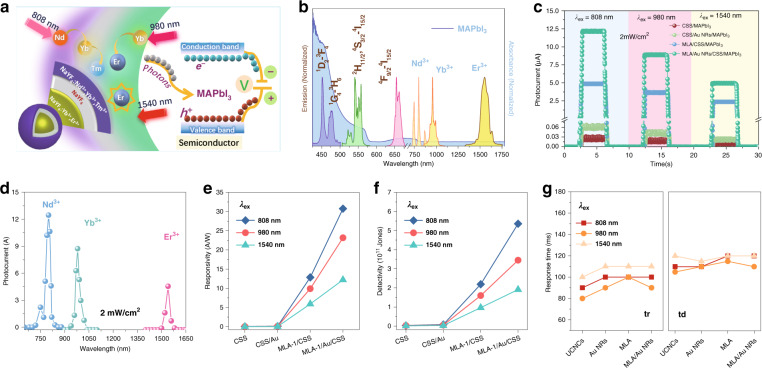
Cascading upconversion amplification for multispectral narrowband NIR photodetection. **a** Schematic illustration of the photodetector device’s mechanism. **b** Absorption of MAPbI_3_ films, Nd^3+^, Yb^3+^, and Er^3+^ and emission spectra from ^1^D_2_ → ^3^F_4_, ^1^G_4_ → ^3^H_6_, ^2^H_11/2_, ^4^S_3/2_ → ^4^I_15/2_, and ^4^F_9/2_ → ^4^I_15/2_transtions in CSS UCNCs. **c** On–off switching currents of CSS/MAPbI_3_, Au NR/CSS/MAPbI_3_, MLA/CSS/MAPbI_3_, and MLA/Au NR/CSS/MAPbI_3_ under 808, 980, and 1540 nm excitation at a power density of 2 mW cm^−2^. **d** Photocurrent response of the MLA/Au NR/CSS/MAPbI_3_ device under irradiance with 650–1650 nm light. **e**–**g**, Responsivity, detectivity, and response times of CSS/MAPbI_3_, Au NR/CSS/MAPbI_3_, MLA/CSS/MAPbI_3_, and MLA/Au NR/CSS/MAPbI_3_ devices under 808, 980, and 1540 nm light irradiation

Encouraged by the very large UCL enhancement for multiple-band excitation achieved by integration of the dielectric superlensing effect and the plasmonic effect, we designed and fabricated multiband-responsive NIR PDs using the optimized MLA-1/Au NR/UCNC (abbreviated as MLA/Au NR/CSS below) hybrid film as a key component. The PD was composed of silver electrodes, a high-quality MAPbI_3_ film acting as the photon-to-current material^40^, and an MLA/Au NR/CSS hybrid film, as illustrated in Supplementary Fig. S9. In the fabrication, the MAPbI_3_ film (~200 nm in thickness) was self-assembled onto the MLA/Au NR/CSS composite structure using a spin-coating method (Supplementary Fig. S10), followed by the deposition of the silver electrodes on the MAPbI_3_ film, indicating its great bendability and flexibility^21,41^. Other reference PDs were also fabricated, all with the CSS UCNCs as the light conversion materials. The working mechanism of the PD is depicted in Fig. 3a, b. Briefly, the Nd^3+^, Yb^3+^, and Er^3+^ ions in the CSS UCNCs maximally absorb the incident NIR photons at approximately 808, 980, and 1540 nm, respectively, and convert them to visible light in the spectral range of 400–700 nm through photon upconversion processes. The upconverted light can be efficiently absorbed by the perovskite MAPbI_3_, which has a narrow band gap (~800 nm), thereby producing a photocurrent.

The performances of all the fabricated PDs were carefully characterized and compared. Figure 3c presents the typical on/off photocurrent-time (*I–t*) response curves of the devices made of pristine CSS UCNCs, Au NR/CSS, MLA/CSS, or MLA/Au NR/CSS under 808, 980 or 1540 nm illumination with an incident light intensity of 2 mW cm^−2^. Compared to the photocurrent of the PD made of pristine CSS UCNCs, the photocurrents of other PDs were all significantly increased, well in agreement with the UCL enhancements for the different cases shown in Fig. 2. The photocurrents reached 12, 9, and 4.6 µA in MLA/Au NR/CSS PDs for 808, 980, and 1540 nm, while they were 0.03, 0.023, and 0.01 µA in pristine PDs under corresponding excitations, respectively. The amplification factors of the photocurrent were estimated to be 410-fold for 808 nm, 390-fold for 980 nm, and 460-fold for 1540 nm light detection. Figure 3d shows the wavelength-dependent photoresponse curves from 650–1650 nm. Three narrow bands in the NIR I and II regions with full widths at half maxima of 20~30 nm are identified, corresponding to the following 4*f*-4*f* transitions: ^4^I_9/2_ → ^4^F_3/2_ for the Nd^3+^ ions, ^2^F_7/2_ → ^2^F_5/2_ for the Yb^3+^ ions (with some weak bands for ^4^I_15/2_ → ^4^I_13/2_ of the Er^3+^ ions), and ^4^I_15/2_ → ^4^I_13/2_ for the Er^3+^ ions. Such selective photodetection of several supernarrow bands cannot be realized in other types of materials, which may open possibilities for highly encrypted communication. Three representative parameters, photoresponsivity (*R*), detectivity (D*) and external quantum efficiency (*EQE*), were employed to further evaluate the performance of the PDs. *R* represents the photocurrent (*I*_ph_) generated per unit of incident power, D* the ability to detect a weak signal from a noisy environment, and *EQE* the number of carriers produced in the external circuit for each absorbed incident photon^42^. They are defined by the following equations:

1 $$R = \frac{{I_{{\mathrm{light}}} - I_{{\mathrm{dark}}}}}{{PS}}$$

2 $$D \ast = \frac{R}{{\left( {2eI_{{\mathrm{dark}}}/S} \right)^{\frac{1}{2}}}}$$

3 $$EQE = R\frac{{hc}}{{\lambda e}}$$

where *I*_light_ and *I*_dark_ are the photocurrents of PDs under light illumination and in the dark, respectively; *P* represents the input light intensity, *S* represents the effective illuminated area; *h* is Planck’s constant, *c* is the velocity of light, *λ* represents the wavelength of incident light, and *e* is the elementary charge. As shown in Fig. 3e–f and Figure S11, the *R*, D*, and *EQE* of PDs all stand out as increased in the MLA/Au NR/CSS PD. Specifically, the *R*, D* and *EQE* values of the MLA/Au NR/CSS PD are 30.73, 23.15, and 12.20 A W^−1^; 5.36 × 10^11^ Jones, 3.45 × 10^11^ Jones, and 1.92 × 10^11^ Jones; and 4726%, 2935%, and 984% for 808, 980, and 1540 nm light detection, respectively. In contrast, they are 0.07, 0.05, and 0.03 A W^−1^; 3.5 × 10^9^ Jones, 2.5 × 10^9^ Jones, and 1.5 × 10^9^ Jones; and 10.6%, 6.4%, and 2.2% for the PD fabricated with the pristine CSS UCNC film. Additional technical details are presented in Supplementary Table I. Excitingly, *R*, D* and *EQE* are amplified 440-fold, 150-fold, and 450-fold for 808 nm, 460-fold, 140-fold, 460-fold for 980 nm, 410-fold, 130-fold, and 450-fold for 1540 nm light detection, respectively. Accordingly, the photodetection thresholds for the MLA/Au NR/CSS PD were largely improved and reached below 0.01–0.03 mW cm^−2^ for 808, 980, and 1540 nm light, more than two orders of magnitude lower than those for the pristine PD (Supplementary Fig. S12). The linear dynamic range (LDR) is another critical photodetector metric and is defined in dB as the range of linear responsivity^43^. To test the linearity of the PDs, we measured the photocurrent as a function of power density for different excitation wavelengths, as shown in Supplementary Fig. S13. The estimated LDR values are 51, 50, and 50 dB under 808, 980, and 1540 nm illumination, respectively. A logarithmic plot of the photocurrent light intensity (Supplementary Fig. S13) shows that within the range of 0.03–2 mW cm^−2^, the photocurrent has a superlinear response owing to the two-photon or multiple-photon absorption of UCNCs. In the range of 2–300 mW cm^−2^, the photocurrent response becomes sublinear with increasing incident light intensity. The fractional power density dependence is considered to be associated with the complex behaviors of upconversion processes and the generation of charge carriers in the PDs^44,45^. Therefore, this sublinear phenomenon can be explained by the co-interaction of the pump saturation effect of UCL and the electron-hole recombination loss in the MAPbI_3_ layer with increasing illumination power^19,46–48^. The *I–V* characteristics of MLA/Au NR/CSS/MAPbI_3_ PDs at different illumination light intensities at 808, 980 and 1540 nm are provided in Supplementary Figs. S14 and S15. Furthermore, the stability of MLA/Au NR/CSS/MAPbI_3_ PDs was examined, as displayed in Supplementary Fig. S16. The photocurrent of MLA/Au NR/CSS/MAPbI_3_ PDs decreases by 45%, 46%, and 45% after 90 days under illumination at 808, 980, and 1540 nm, respectively.

The photon response times (rise and decay) of a PD are important technical parameters, and generally, a fast response is desired. In our designed UCNC-based PDs, the response times are fundamentally determined by the temporal properties of the UCL emitted by the UCNCs, with some additional delay after the integration of the whole device. A fascinating property of UCNCs is that their luminescence kinetics can be flexibly altered by changing the concentration of doping ions. To achieve fast UCL kinetics, we engineered the UCNCs by varying the Nd^3+^ concentration from 10% to 40%, the Yb^3+^ concentration from 10% to 40%, and the Er^3+^ concentration from 1% to 8%. Similarly sized CSS UCNCs were obtained after the doping concentrations were varied (Supplementary Fig. S17). This strategy largely altered the UCL kinetics, both the rise and decay times (Supplementary Fig. S18). Accordingly, the response times (rise and decay) are altered by approximately 50% (Supplementary Fig. S18, Supplementary Table II). In addition, the photocurrents under 808, 980, and 1540 nm illumination also change by 20–36% when the doping concentrations are varied (Supplementary Fig. S19). Similarly, the *R*, D*, and *EQE* values of the PDs vary noticeably as well (Supplementary Table II). The photon-response times, including the rise (*t*_rise_) and decay times (*t*_decay_), of PDs fabricated with the optimized CSS UCNCs that were extracted from the dynamic response curves of photocurrents of various PDs under 808, 980, or 1540 nm light illumination (Supplementary Fig. S20) are presented in Fig. 3g. All the PDs exhibit fast response times in the range of 80–120 ms, and the integration with both Au NRs and MLAs has a slight influence on the photon-response times of the device. In contrast to other reported NIR PDs (Table 1), multispectral super-narrow band NIR PDs are reported here for the first time and exhibit much better performances in almost all aspects. In particular, the photoresponsivity *R* and detectivity D* are remarkably increased by one to three orders of magnitude, and the response time is also significantly curtailed.

**Table 1:** Comparison of device performance of our and other NIR PDs.

Material and structure	Spectral range [nm]		Responsivity [A W^−1^]	Detectivity [Jones]	EQE	Response time [ms]	Ref.
Commercial Ge	800–1800		0.85	3.0 × 10^11^			^50^
Graphene/Si	850–900		0.435	7.69 × 10^9^		>1	^55^
PbPc/CuPc	600–1000	808	2.3	4.0 × 10^11^			^56^
C_60_/PTCDA: AlClPcPbPc	300–900	850	0.33	10^9^–10^10^			^57^
Bi_2_Te_3_-SnSe-Bi_2_Te_3_	370–808		5.5	6.0 × 10^10^	18.33		^58^
Perovskite/Conjugated-polymer composite	UV-Vis-NIR	735	~0.0225	~1.3 × 10^10^	~0.04		^52^
		835	~0.01	~5.2 × 10^9^	~0.02	30	
		937	0.0055	3.2 × 10^9^	~0.01		
MoS_2_/UCNPs	633–1342		1.0 × 10^–4^	1.0 × 10^–4^			^59^
UCNPs/Graphene/GaAs	980		0.00597	1.1 × 10^11^			^60^
Black Phosphorus	400–1700	1200	3.5 × 10^–4^			0.04	^61^
MLA/Au NRs/UCNCs/ MAPbI_3_	NIR	808	30.73	5.36 × 10^11^	47.26	100	Our work
		980	23.15	3.45 × 10^11^	29.35	90	
		1540	12.20	1.91 × 10^11^	9.84	110	

**Fig. 4:**
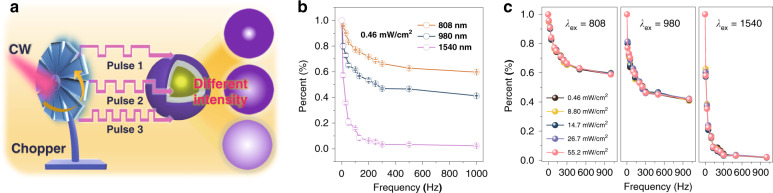
Modulation frequency response for selective multispectral photodetections. **a** Schematic drawing illustrating the mechanism for the selective multispectral dependence of the emission on the excitation frequency. **b**, **c** Relative UCL intensity upon changing the excitation light frequency (**b**) and simultaneously varying the excitation power density (**c**)

Discriminating the wavelength of the incident light is very important for a multiband photodetector. From our previous studies, we realized that each UCNC constitutes a kinetic optical system formed by many coupled optical centers (lanthanide ions) that can have fingerprint responses to certain optical stimuli, e.g., excitation wavelength, excitation intensity, and excitation modulation^28,29,49^. Considering that the CSS UCNCs have different response speeds at different excitation wavelengths, rooted in differences of the photon upconversion pathways, we exploited the possibility of distinguishing excitation wavelengths using the UCNC’s response to the excitation modulation frequency, a Fourier transformation to time. Experimentally, a mechanical chopper was placed in the optical path of an incident continuous-wave (CW) light beam to generate square-wave (SW) output (Fig. 4a), the frequency of the square wave was varied in the range of 0–1000 Hz, and the corresponding UCL intensity was recorded. Figure 4b and Supplementary Fig. S21 display the UCL intensities and spectra of the CSS NCs at different frequencies of incident light. The UCL intensity remarkably decreases for all three excitation wavelengths (808, 980, and 1540 nm) at the same power density (0.46 mW cm^−2^). Intriguingly, the decrease extent of the UCL intensity and rates are quite different and thus well distinguishable. Moreover, the trend of changes for all incident wavelengths is insensitive to the excitation power in a broad range (0.46–55.2 mW cm^−2^), as shown in Fig. 4c. Thus, it can be concluded that the incident wavelength (single wavelength, CW) can be well identified by examining the response of the CSS UCNCs to the excitation modulation frequency. The chopping of the incident beam somewhat increases the complexity in wavelength analysis and limits the incident light by repetitively blocking its access to the PD. However, chopping is relatively easily implemented with a turning wheel, and the blocked light can easily be reflected and fed into a parallel PD, thereby not losing half of the incident light to be detected in this approach.

## Discussion

We designed and synthesized NaYF_4_:Yb^3+^, Er^3+^@NaYF_4_@NaYF_4_:Yb^3+^, Nd^3+^, Tm^3+^ core-shell-shell (CSS) upconversion nanocrystals (UCNCs) that are excited by 808, 980, and 1540 nm light and generate visible emissions. By using a novel cascade optical field modulation strategy through integrating the superlensing effect of dielectric microlens arrays (MLAs) and the plasmonic effect of Au NRs, significant upconversion luminescence (UCL) enhancements from the UCNCs were obtained; specifically, signals were increased by factors of 2.4 × 10^4^, 2.2 × 10^4^, and 1.6 × 10^4^ for 808, 980, and 1540 nm excitation, respectively. In comparison, the LSPR effect can typically enhance UCL by one order of magnitude, while the superlensing effect can lead to UCL enhancement by two or three orders of magnitude when the same routine and easily obtained nano-/microstructures are used^23–25^. Photodetectors (PDs) were built based on the synthesized CSS UCNCs adopting the cascade optical field modulation strategy for UCL, and they achieve selective detection of three narrow spectral bands in the near-infrared (NIR) region. The PDs exhibit extremely high responsivities of 30.73, 23.15, and 12.20 A W^−1^ and detectivities of 5.36 × 10^11^, 3.45 × 10^11^, and 1.92 × 10^11^ Jones for 808, 980, and 1540 nm light detection, respectively, which are comparable to or well beyond those of other types of NIR PDs, such as commercial Ge-based PDs (Table 1)^50^. The PDs also exhibit short response times in the range of 80–100 ms. Importantly, the incident light wavelength can be well distinguished by a proposed novel approach, i.e., examining responses to the excitation modulation frequency. This work highlights new concepts to conquer the relatively high pumping threshold of UCNCs, enabling the building of high-photoresponsivity and high-detectivity multiband-responsive and distinguishable photodetectors on top of them and can also stimulate other applications of upconversion nanotechnology.

## Materials and methods

### Synthesis of core–shell–shell UCNCs

The designed NaYF_4_:Yb^3+^, Er^3+^@NaYF_4_@NaYF_4_:Yb^3+^, Nd^3+^, Tm^3+^ UCNCs were prepared following a previously reported solvothermal method^51^. In a typical procedure, a 100 mL three-neck flask charged with oleic acid (6 mL) and octadecene (15 mL), YCl_3_·6H_2_O, YbCl_3_·6H_2_O and ErCl_3_·6H_2_O at the molar ratio 78/20/2 received 1 mmol of total lanthanide. After stirring for 1 h at 160 °C in an oil bath, a lanthanide mixture was formed. Then, the resultant complexes were cooled to room temperature. A solution of NaOH (2.5 mmol) and NH_4_F (4 mmol) in methanol (6 mL) was added to the above mixture, which was then stirred for 1 h. The mixture was then transferred into a heating mantle and heated at 300 °C for 1.5 h. To avoid oxidation of the mixture, the whole experiment was carried out in a nitrogen atmosphere. After cooling to room temperature, the resulting UCNCs were collected and washed by centrifugation with absolute ethanol-cyclohexane (1:1 v/v) several times and redispersed in 5 mL cyclohexane solution for subsequent shell growth. In the preparation of NaYF_4_:Yb, Er@NaYF_4_ core-shell UCNCs, a shell precursor solution containing 1 mmol of YCl_3_·6H_2_O was prepared using the same process as described above and cooled to 80 °C. Then, the as-prepared core UCNC suspension was added. After 45 min, the resultant mixture was cooled to room temperature, and a methanol solution (6 mL) of NaOH (2.5 mmol) and NH_4_F (4 mmol) was subsequently added. After stirring for another 30 min at 30 °C, this mixture was heated to 80 °C for 30 min before being heated to 300 °C. After reaction completion in 1.5 h, the as-prepared core-shell UCNCs were collected and washed by the procedure described above. The synthesis of core-shell-shell UCNCs followed the same procedure as that of core-shell UCNCs except that the shell precursor solution had a Y^3+^/Yb^3+^/Nd^3+^/Tm^3+^ ratio of 59.8/20/20/0.2.

### Synthesis of gold nanorods

The Au NRs were synthesized according to an improved seeded growth method using binary surfactant mixtures^23^. First, the seed solution for Au NR growth was prepared as follows: 5 mL of 0.2 M hexadecyltrimethylammonium bromide (CTAB) solution was mixed with 5 mL of 0.5 mM HAuCl_4_. Afterwards, 0.6 mL of fresh 0.01 M NaBH_4_ was diluted to 1 mL with deionized water and then added to the Au (III)-CTAB solution under vigorous stirring (1600 rpm). After continuous stirring for 3 min, the color of the mixture changed from yellow to dark brown. The seed solution was aged at room temperature for 30 min before use.

For the preparation of a growth solution, 0.025 g of NaOL dissolved in 5 mL of deionized water was reacted with 0.137 g of CTAB at room temperature and then mixed with 18 μL of 100 mM AgNO_3_ solution. After that, 5 mL of 1 mM HAuCl_4_ solution and 72 μL of HCl (37 wt.% in water, 12.1 M) were added, which was followed by vigorous stirring for 1.5 h. After another 0.5 h with gentle stirring, 1.25 mL of 0.064 M ascorbic acid (AA) was added to the solution with vigorous stirring for 30 s. A small amount of seed solution (0.2 mL) was then injected into the growth solution. Finally, the resultant mixture was stirred for 30 s and then left undisturbed for 12 h at room temperature for NR growth. The final products were isolated and washed by centrifugation with deionized water at 7000 rpm for 30 min, followed by removal of the supernatant. Note that changing the amount of seed solution from 0.2–0.25 mL, HCl from 0.072–0.1 mL, and NaOL from 0.025–1.234 g can control the aspect ratio of Au NRS.

### Fabrication of MLA/Au NR/CSS/MAPbI_3_ hybrid PDs

First, an MLA film was treated with ozone for 60 min, and then Au NR and CSS UCNC layers were sequentially fabricated on the MLA substrate by a spin-coating method at 2500 rpm for 30 s and at 2500 rpm for 30 s, respectively. A perovskite film was fabricated on the MLA/Au NR/CSS hybrids under a nitrogen environment by a typical two-step method^52^. Then, the MLA/Au NR/CSS hybrids were treated with ozone for 120 min. During the first step, the prepared MAPbI_3_ perovskite precursor solution was obtained by mixing PbI_2_ and MAI in dimethyl sulfoxide solution. Afterwards, the MAPbI_3_ perovskite precursor solution and 100 μL of chlorobenzene were kept on a spinning MLA/Au NR/CSS substrate at 1200 and 4000 rpm for 12 and 30 s, respectively. The MAPbI_3_ films were formed after annealing at 100 °C for 10 min.

### Characterization

The morphologies of all samples were recorded by a Hitachi H-8100IV transmission electron microscope under an acceleration voltage of 200 kV or by a scanning electron microscope. The purities and phase structures of the products were characterized by X-ray diffraction. UV/vis-NIR absorption spectra of the products were measured with a Shimadzu UV-3600PC UV/vis-NIR scanning spectrophotometer in the range of 300 to 2500 nm. The morphologies of different MLA samples were determined by an Asylum MFP-3D atomic force microscope. For film sample characterization, power-dependent UCL spectra were measured by a home-built Olympus IX73P2F fluorescence microscope and a Princeton Instruments SP2300 coupled with 808, 980 and 1540 nm diode lasers for excitation. For luminescence kinetics measurements, a Tektronix AFG1022 function generator was used to modulate the laser source. The performance measurements of the devices were carried out on a Tektronix 2400 system.

### Numerical simulation

Simulation of the light converging effect of MLAs and the plasmonic effect of Au NR films was performed by a three-dimensional finite-difference time-domain (3D-FDTD) method with periodic boundary conditions. The excitation light was set as a plane wave at a wavelength of 808, 980 or 1540 nm. The refractive indices of β–NaYF_4_ UCNCs and MLAs are approximately 1.468 and 1.570 at 808 nm, 1.466 and 1.565 at 980 nm, and 1.464 and 1.561 at 1540 nm, respectively^53,54^.

### Modulation frequency response and photocurrent measurements

A mechanical chopper (Model SR540 chopper controller) was placed in the optical path of the incident CW laser beam to generate square-wave light irradiation. The frequency of the chopper was varied in the range of 0–1000 Hz, and the resulting UCL spectrum and intensity were recorded (Andor SR750-B1). The photocurrents of PDs were measured by a Tektronix 2400 and analyzed by Lab Tracer 2.9 applications.

## Supplementary information

Supplementary information

## References

[1] Velusamy DB (2015). Flexible transition metal dichalcogenide nanosheets for band-selective photodetection. Nat. Commun..

[2] Wang T (2019). A crystal-growth boundary-fusion strategy to prepare high-quality MAPbI_3_ films for excellent Vis-NIR photodetectors. Nano Energy.

[3] Armin A (2015). Narrowband light detection via internal quantum efficiency manipulation of organic photodiodes. Nat. Commun..

[4] Zou WY (2018). Skin color-specific and spectrally-selective naked-eye dosimetry of UVA, B and C radiations. Nat. Commun..

[5] Luther JM (2011). Localized surface plasmon resonances arising from free carriers in doped quantum dots. Nat. Mater..

[6] Zhou N (2017). Narrowband spectrally selective near-infrared photodetector based on up-conversion nanoparticles used in a 2D hybrid device. J. Mater. Chem. C..

[7] Xie C (2018). Graphene/semiconductor hybrid heterostructures for optoelectronic device applications. Nano Today.

[8] Dai MJ (2018). A dual-band multilayer InSe self-powered photodetector with high performance induced by surface Plasmon resonance and asymmetric schottky junction. ACS Nano.

[9] Park HG (2008). A wavelength-selective photonic-crystal waveguide coupled to a nanowire light source. Nat. Photonics.

[10] Lee CH (2014). Atomically thin p-n junctions with van der Waals heterointerfaces. Nat. Nanotechnol..

[11] Li JZ (2019). Self-trapped state enabled filterless narrowband photodetections in 2D layered perovskite single crystals. Nat. Commun..

[12] Zheng J (2013). Seven-photon ultraviolet upconversion emission of Er^3+^ induced by 1,540-nm laser excitation. Appl. Phys. B.

[13] Liu XD (2018). Recent advances in organic near-infrared photodiodes. J. Mater. Chem. C..

[14] Sobhani A (2013). Narrowband photodetection in the near-infrared with a Plasmon-induced hot electron device. Nat. Commun..

[15] Cao F (2020). Bionic detectors based on Low-Bandgap inorganic Perovskite for selective NIR-I photon detection and imaging. Adv. Mater..

[16] Xu CT (2013). Upconverting nanoparticles for pre-clinical diffuse optical imaging, microscopy and sensing: current trends and future challenges. Laser Photonics Rev..

[17] Liu KC (2016). A flexible and superhydrophobic upconversion-luminescence membrane as an ultrasensitive fluorescence sensor for single droplet detection. Light Sci. Appl..

[18] Wang F (2018). Microscopic inspection and tracking of single upconversion nanoparticles in living cells. Light Sci. Appl..

[19] Ji YN (2019). Semiconductor plasmon enhanced monolayer upconversion nanoparticles for high performance narrowband near-infrared photodetection. Nano Energy.

[20] Han JW (2019). Enhanced outcoupling in down-conversion white organic light-emitting diodes using imprinted microlens array films with breath figure patterns. Sci. Technol. Adv. Mater..

[21] Liu QY (2019). Microlens array enhanced upconversion luminescence at low excitation irradiance. Nanoscale.

[22] He JJ (2017). Plasmonic enhancement and polarization dependence of nonlinear upconversion emissions from single gold nanorod@SiO_2_@CaF_2_:Yb^3+^,Er^3+^ hybrid core–shell–satellite nanostructures. Light Sci. Appl..

[23] Yin Z (2016). Local field modulation induced three-order upconversion enhancement: combining surface Plasmon effect and photonic crystal effect. Adv. Mater..

[24] Li DY (2018). Plasmonic photonic crystals induced two-order fluorescence enhancement of blue perovskite nanocrystals and its application for high-performance flexible ultraviolet photodetectors. Adv. Funct. Mater..

[25] Yu XC (2018). Atomically thin noble metal dichalcogenide: a broadband mid-infrared semiconductor. Nat. Commun..

[26] Liang LL (2019). Upconversion amplification through dielectric superlensing modulation. Nat. Commun..

[27] Zhan QQ (2015). Tens of thousands-fold upconversion luminescence enhancement induced by a single gold nanorod. Laser Photonics Rev..

[28] Liu HC (2018). Photon upconversion kinetic nanosystems and their optical response. Laser Photonics Rev..

[29] Bagheri N (2019). Change in the emission saturation and kinetics of upconversion nanoparticles under different light irradiations. Optical Mater..

[30] Wen SH (2018). Advances in highly doped upconversion nanoparticles. Nat. Commun..

[31] Zhang JH (2015). Observation of efficient population of the red-emitting state from the green state by non-multiphonon relaxation in the Er^3+^-Yb^3+^ system. Light Sci. Appl..

[32] Huang XY, Lin J (2015). Active-core/active-shell nanostructured design: an effective strategy to enhance Nd^3+^/Yb^3+^ cascade sensitized upconversion luminescence in lanthanide-doped nanoparticles. J. Mater. Chem. C..

[33] Wiesholler LM (2019). Yb,Nd,Er-doped upconversion nanoparticles: 980 nm *versus* 808 nm excitation. Nanoscale.

[34] Ding MY (2018). Nd^3+^/Yb^3+^ cascade-sensitized single-band red upconversion emission in active-core/active-shell nanocrystals. Nanotechnology.

[35] Fan XF, Zheng WT, Singh DJ (2014). Light scattering and surface plasmons on small spherical particles. Light Sci. Appl..

[36] Su YH (2012). Surface Plasmon resonance of layer-by-layer gold nanoparticles induced photoelectric current in environmentally-friendly Plasmon-sensitized solar cell. Light Sci. Appl..

[37] Liu AH (2017). Gold nanostructures with near-infrared plasmonic resonance: synthesis and surface functionalization. Coord. Chem. Rev..

[38] Fischer S (2016). Enhanced upconversion quantum yield near spherical gold nanoparticles-a comprehensive simulation based analysis. Opt. Express.

[39] Homann C (2018). NaYF_4_:Yb,Er/NaYF_4_ core/shell nanocrystals with high upconversion luminescence quantum yield. Angew. Chem. Int. Ed..

[40] Gu LL, Fan ZY (2017). Perovskite/organic-semiconductor heterojunctions for ultrasensitive photodetection. Light Sci. Appl..

[41] Jin JJ (2017). Enhanced performance of perovskite solar cells with zinc chloride additives. ACS Appl. Mater. Interfaces.

[42] Lin T, Wang JZ (2019). Strategies toward high-performance solution-processed lateral photodetectors. Adv. Mater..

[43] Shen L (2016). A self-powered, sub-nanosecond-response solution-processed hybrid perovskite photodetector for time-resolved photoluminescence-lifetime detection. Adv. Mater..

[44] Ouyang WX, Teng F, Fang XS (2018). High performance BiOCl nanosheets/TiO_2_ nanotube arrays heterojunction UV photodetector: the influences of self-induced inner electric fields in the BiOCl nanosheets. Adv. Funct. Mater..

[45] Mazzeo G (2006). Deep ultraviolet detection dynamics of AlGaN based devices. Appl. Phys. Lett..

[46] Zhang XH (2017). Perovskite-erbium silicate nanosheet hybrid waveguide photodetectors at the near-infrared telecommunication band. Adv. Mater..

[47] Wu D (2012). Device structure-dependent field-effect and photoresponse performances of p-type ZnTe:Sb nanoribbons. J. Mater. Chem..

[48] Li CL (2020). Ultrafast and broadband photodetectors based on a perovskite/organic bulk heterojunction for large-dynamic-range imaging. Light Sci. Appl..

[49] Koppens FHL (2014). Photodetectors based on graphene, other two-dimensional materials and hybrid systems. Nat. Nanotechnol..

[50] Saran R, Curry RJ (2016). Lead sulphide nanocrystal photodetector technologies. Nat. Photonics.

[51] Li ZQ, Zhang Y, Jiang S (2008). Multicolor core/shell-structured upconversion fluorescent nanoparticles. Adv. Mater..

[52] Chen S (2016). A flexible UV-Vis-NIR photodetector based on a perovskite/conjugated-polymer composite. Adv. Mater..

[53] Sokolov VI (2015). Determination of the refractive Index of β-NaYF_4_/Yb^3+^/Er^3+^/Tm^3+^ nanocrystals using spectroscopic refractometry. Opt. Spectrosc..

[54] https://refractiveindex.info/?shelf=organic&book=poly(methyl_methacrylate)&page=Szczurowski.

[55] An XH (2013). Tunable graphene-silicon heterojunctions for ultrasensitive photodetection. Nano Lett..

[56] Huang FB (2017). Improved performance of lead phthalocyanine phototransistor by template inducing effect based on optimized-thickness copper phthalocyanine layers. Synth. Met..

[57] Huang FB (2017). Towards high performance broad spectral response fullerene based photosensitive organic field-effect transistors with tricomponent bulk heterojunctions. Carbon.

[58] Yao JD, Zheng ZQ, Yang GW (2017). All-layered 2D optoelectronics: a high-performance UV-vis-NIR broadband SnSe Photodetector with Bi_2_Te_3_ topological insulator electrodes. Adv. Funct. Mater..

[59] Zhang YW (2018). Extending the spectral responsivity of MoS_2_ phototransistors by incorporating up-conversion microcrystals. Adv. Optical Mater..

[60] Wu JH (2018). Enhanced performance of a graphene/GaAs self-driven near-infrared photodetector with upconversion nanoparticles. Nanoscale.

[61] Yuan HT (2015). Polarization-sensitive broadband photodetector using a black phosphorus vertical p-n junction. Nat. Nanotechnol..

