# Supplementary material for: Huge upconversion luminescence enhancement by a cascade optical field modulation strategy facilitating selective multispectral narrow-band near-infrared photodetection
Source: Light Sci Appl. 2020 Oct 30;9:184. doi: 10.1038/s41377-020-00418-0 (PMC7603315; doi:10.1038/s41377-020-00418-0)
Supplement: Supplementary file 1 — Supplementary information [file 41377_2020_418_MOESM1_ESM.docx]

**Supplementary Information**

**Huge upconversion luminescence enhancement by a cascade optical field modulation strategy facilitating selective multispectral narrow-band near-infrared photodetection**

Yanan Ji^1^, Wen Xu^1*^, Nan Ding^1^, Haitao Yang^1^, Hongwei Song^1*^, Qingyun Liu^2^, Hans Ågren^2^, Jerker Widengren^3^, Haichun Liu^2,3*^

^1^ State Key Laboratory of Integrated Optoelectronics, College of Electronic Science and Engineering, Jilin University, 130012 Changchun, China

^2^ Department of Theoretical Chemistry and Biology, KTH Royal Institute of Technology, SE-106 91 Stockholm, Sweden

^3^ Department of Applied Physics, KTH Royal Institute of Technology, SE-106 91 Stockholm, Sweden

E-mail: [wen_xu@jlu.edu.cn; songhw@jlu.edu.cn](mailto:wen_xu@jlu.edu.cn;%20songhw@jlu.edu.cn); haichun@kth.se

**Supplementary Figure 1.** TEM images and size distribution of (a) NaYF_4_: 20%Yb^3+^, 2%Er^3+^ and (b) NaYF_4_: 20%Yb^3+^, 2%Er^3+^@NaYF_4_ UCNCs.

**Supplementary Figure 2.** a, S-TEM image and elemental mapping of a single NaYF_4_: 20%Yb^3+^, 2%Er^3+^@NaYF_4_@NaYF_4_: 20%Yb^3+^, 20%Nd^3+^, 0.2%Tm^3+^ CSS NC. b-d, Corresponding line-scan elemental mapping curves.

**Supplementary Figure 3.** XRD characterizations of the as-prepared hexagonal UCNCs.

**Supplementary Figure 4.** Schematic diagram of the energy levels of Tm^3+^, Yb^3+^, and Nd^3+^ ions and its additional energy transfer processes under 808 nm excitation.

Cross relaxation Nd×Tm,^1^

1. Nd(^4^F_3/2_) + Tm(^3^H_4_) → Nd(^4^I_11/2_) + Tm(^1^G_4_)；
2. Nd(^4^F_3/2_) + Tm(^3^F_4_) → Nd(^4^I_11/2_) + Tm(^3^F_2_);

Energy transfer Tm-Nd,^2^

1. Tm(^3^H_4_)→ Nd(^4^F_3/2_);

Cross relaxation Nd×Nd,^3^

1. Nd(^4^F_3/2_) + Nd(^4^I_9/2_) → Nd(^4^I_15/2_) + Nd(^4^I_15/2_).

**Supplementary Figure 5.** a-c, TEM images of (a) with the aspect ratio is about 2.94, (b) is about 4.28 and (c) is about 5.84. d, LSPR absorbance spectrum of as-prepared Au NRs solutions, and their LSPR peak maxima are 809, 989, and 1152 nm, respectively.

**Supplementary Figure 6.** a-d, AFM images and thickness of Au NRs films, monolayer CSS UCNCs films, single CSS nanoparticle, and Au NRs/CSS films.

**Supplementary Figure 7.** Decay curves for the (a) CSS, Au NRs/CSS, MLA-1/CSS and MLA-1/Au NRs/CSS hybrids; (b) MLA-2/CSS and MLA-3/CSS films. All the experiments were performed under 980 nm excitation. c, Decay time constans of ^1^G_4_-^3^H_6_, ^4^S_3/2_-^4^I_15/2_, ^4^F_9/2_-^4^I_15/2_ transitions of CSS, Au NRs/CSS, MLAs/CSS, and MLAs/Au NRs/CSS.

**Supplementary Figure 8.** FDTD simulations of the electric field distribution of (a) MLA-1, (b) MLA-2, (c) MLA-3, (d) Au NRs/MLA-1 hybrids and (e) Au NRs under NIR excitation light. Note that 808, 980, and 1540 nm plane wave light source are used for excitation, respectively.

**Supplementary Figure 9.** Schematic illustration of selectively multispectral narrowband NIR PDs at 808, 980, and 1540 nm based on the MLA/Au NRs/CSS/MAPbI_3_ hybrid structure.

**Supplementary Figure 10.** a, XRD pattern of MAPbI_3_. b-d SEM characterization: the top view image (b), cross section image (c) of the MAPbI_3_ perovskite films and the cross section image (d) of the MLA/Au NRs/CSS/MAPbI_3_ composites structure.

**Supplementary Figure 11.** EQE of CSS, Au NRs/CSS, MLA/CSS, and MLA/Au NRs/CSS devices.

**Supplementary Figure 12.** a-c, On-off switching currents of CSS/MAPbI_3_ and MLA/Au NRs/CSS/MAPbI_3_ at lowest detection excitation power, under the 808 nm (a), 980 nm (b) and 1540 nm(c) excitation.

**Supplementary Figure 13.** Photocurrent as a function of light intensity for the (a) 808 nm, (b) 980 nm, and (c) 1540 nm illumination.

**Supplementary Figure 14.** *I-V* characteristics of MLAs/Au NRs/CSS/MAPbI_3_ PDs as a function of incident light intensity under illumination with 808 nm (a), 980 nm (b) and 1540 nm (c), respectively.

**Supplementary Figure 15.** The corresponding light power density-dependent photoresponse separately under 808 nm (a), 980 nm (b), and 1540 nm (c) laser illumination at a bias of 1 V.

**Supplementary Figure 16.** The stability of MLAs/Au NRs/CSS/MAPbI_3_ PDs.

**Supplementary Figure 17.** TEM characterizations of NaYF_4_: 20%Yb, 2%Er@NaYF_4_@*x*%Nd, 20%Yb, 0.02%Tm CSS UCNCs with separately varying Nd^3+^ concentration from 10% to 40% (a-d), NaYF_4_: *y*%Yb, 2%Er@NaYF_4_@20%Nd, 20%Yb, 0.02%Tm CSS UCNCs with separately varying Yb^3+^ concentration from 10% to 40% (e-h), NaYF_4_: 20%Yb, *z*%Er@NaYF_4_@20%Nd, 20%Yb, 0.02%Tm CSS UCNCs with separately varying Er^3+^ concentration from 1% to 8% (i-l).

**Supplementary Figure 18.** a-c, The concentration of doping ions dependence decay time. (a) Varying Nd^3+^ concentration from 10% to 40%, (b) Yb^3+^ concentration from 10% to 40%, (c) and Er^3+^ concentration from 1% to 8%, respectively. d-f, *I*-*t* curves of MLA/Au NRs/CSS/MAPbI_3_ device as a function of increasing the Nd^3+^ concentration (d), Yb^3+^ concentration (e) and Er^3+^ concentration (f).

**Supplementary Figure 19.** *I-t* curves of NaYF_~~4~~_: *x*%Yb, *x*%Er@NaYF_4_@*x*%Nd, 20%Yb, 0.02%Tm CSS UCNCs with separately varying Nd^3+^ concentration from 10% to 40%, Yb^3+^ concentration from 10% to 40%, and Er^3+^ concentration from 1% to 8%, excitation source with 2 mW cm^-2^ power density.

**Supplementary Figure 20.** a-c, On-off switching currents of MLA/Au NRs/CSS/MAPbI_3_ at lowest detection excitation power, under the 808 nm (a), 980 nm (b) and 1540 nm (c) excitation.

**Supplementary Figure 21.** Excitation frequency dependence UCL intensity of MLA/Au NRs/CSS films, (a) for 808 nm excitation, (b) for 980 nm excitation and (c) for 1540 nm excitation.

**Supplementary Table I.** The *D**, *R*, *EQE*, and response times of the CSS/MAPbI_3_, Au NRs/CSS/MAPbI_3_, MLA/CSS/MAPbI_3_, MLA/Au NRs/CSS/MAPbI_3_ PDs under separately 808, 980, and 1540 nm irradiation at a power density of 2 mW cm^-2^.

**Supplementary Table II.** The technical data of the MLA/Au NRs/UCNCs/MAPbI_3_ PD.

**References:**

1. Song S. A. *et al.* Upconversion in Nd-Tm-Yb triply doped oxyfluoride glass-ceramics containing CaF_2_ nanocrystals. *J. Lumin.,*152, 75(2014).
2. Chung W. J. et al. Energy transfer process for the blue up-conversion in calcium aluminate glasses doped with Tm^3+^ and Nd^3+^. *J. Am. Ceram. Soc.*, 84, 348(2001).
3. Liang L. L. et al. Designing upconversion nanocrystals capable of 745 nm sensitization and 803 nm emission for deep-tissue imaging. Chem. Eur. J., 22, 10801(2016).
